# CD73 expression defines immune, molecular, and clinicopathological subgroups of lung adenocarcinoma

**DOI:** 10.1007/s00262-020-02820-4

**Published:** 2021-01-08

**Authors:** Pedro Rocha, Ruth Salazar, Jiexin Zhang, Debora Ledesma, Jose L. Solorzano, Barbara Mino, Pamela Villalobos, Hitoshi Dejima, Dzifa Y. Douse, Lixia Diao, Kyle Gregory Mitchell, Xiuning Le, Jianjun Zhang, Annikka Weissferdt, Edwin Parra-Cuentas, Tina Cascone, David C. Rice, Boris Sepesi, Neda Kalhor, Cesar Moran, Ara Vaporciyan, John Heymach, Don L. Gibbons, J. Jack Lee, Humam Kadara, Ignacio Wistuba, Carmen Behrens, Luisa Maren Solis

**Affiliations:** 1 Department of Translational Molecular Pathology, The University of Texas MD Anderson Cancer Center, 2130 West Holcombe Boulevard, Houston, TX 77030 USA; 2 Universidad de Barcelona, Barcelona, Spain; 3 Department of Bioinformatics and Comp Biology, The University of Texas MD Anderson Cancer Center, Houston, TX USA; 4 Thoracic/Head and Neck Medical Oncology, The University of Texas MD Anderson Cancer Center, Houston, TX USA; 5 Thoracic and Cardiovascular Surgery, The University of Texas MD Anderson Cancer Center, Houston, TX USA; 6 The University of Texas MD Anderson Cancer Center, Houston, TX USA

**Keywords:** CD73, Lung adenocarcinoma, Immune profiling, Adenosinergic pathway, PD-L1

## Abstract

**Introduction:**

CD73 is a membrane-bound enzyme crucial in adenosine generation. The adenosinergic pathway plays a critical role in immunosuppression and in anti-tumor effects of immune checkpoint inhibitors (ICI). Here, we interrogated CD73 expression in a richly annotated cohort of human lung adenocarcinoma (LUAD) and its association with clinicopathological, immune, and molecular features to better understand the role of this immune marker in LUAD pathobiology.

**Materials and methods:**

Protein expression of CD73 was evaluated by immunohistochemistry in 106 archived LUADs from patients that underwent surgical treatment without neoadjuvant therapy. Total CD73 (T +) was calculated as the average of luminal (L +) and basolateral (BL +) percentage membrane expression scores for each LUAD and was used to classify tumors into three groups based on the extent of T CD73 expression (high, low, and negative).

**Results:**

*CD73* expression was significantly and progressively increased across normal-appearing lung tissue, adenomatous atypical hyperplasia, adenocarcinoma in situ, minimally invasive adenocarcinoma, and LUAD. In LUAD, BL CD73 expression was associated with an increase in PD-L1 expression in tumor cells and increase of tumor-associated immune cells. Stratification of LUADs based on T CD73 extent also revealed that tumors with high expression of this enzyme overall exhibited significantly elevated immune infiltration and PD-L1 protein expression. Immune profiling demonstrated that T-cell inflammation and adenosine signatures were significantly higher in CD73-expressing lung adenocarcinomas relative to those lacking CD73.

**Conclusion:**

Our study suggests that higher CD73 expression is associated with an overall augmented host immune response, suggesting potential implications in the immune pathobiology of early stage lung adenocarcinoma. Our findings warrant further studies to explore the role of CD73 in immunotherapeutic response of LUAD.

**Supplementary Information:**

The online version contains supplementary material available at 10.1007/s00262-020-02820-4.

## Introduction

Despite significant improvements in treatment, lung cancer remains the leading cause of cancer-related deaths worldwide [1]. Immune checkpoint inhibitors (ICI), as a single agent or in combination with chemotherapy, are increasingly becoming the standard treatment for non-small cell lung cancer (NSCLC), including advanced-stage lung adenocarcinoma (LUAD) [2–5]. Recent studies have shed light on the clinical value of immunotherapy for earlier stage lung tumors including in the neoadjuvant and adjuvant settings [6, 7]. Yet, a limited fraction of NSCLC patients respond to immune checkpoint blockade consisting of anti-PD-1/PD-L1 (Programmed death 1/Programmed death-ligand 1) and CTLA-4 (Cytotoxic T Lymphocyte-associated 4); perhaps warranting the need for other combinatorial immunotherapeutic regimens to potentiate anti-tumor effects of ICI.

Adenosine is generated in the tumor microenvironment owing mainly to the degradation of extracellular ATP [8–10] and NAD +  [11]. Several ectonucleotidases tightly control levels of ATP and Adenosine, such as CD38, CD39, and CD73; among them, CD73 irreversibly converts AMP to Adenosine and was suggested as the rate-limiting enzyme for adenosine formation [12]. Increased adenosine levels permit an immune-tolerant tumor microenvironment by regulating the functions of immune and inflammatory cells such as macrophages, dendritic cells, myeloid-derived suppressor cells, T cells, and natural killer (NK) cells [13]. Adenosine also regulates cancer growth and dissemination by interfering with cell proliferation, apoptosis, and angiogenesis via adenosine receptors expressed on cancer cells and endothelial cells [14–16].

Tumor microenvironment (TME) immunosuppression has emerged as a sentinel mechanism in lung cancer progression and, thus, a viable phenotypic target for treatment [17, 18]. In this context, numerous therapeutic approaches are currently under development with the goal of skewing the TME toward an immune effective phenotype [19]. More recently, in preclinical studies, agents that target the adenosine pathway, including anti-CD73 antibodies and adenosine A2A receptor antagonists, were shown to also attenuate immunosuppression [20, 21].

While CD73 expression in LUAD was noted previously [22], the association of this immune enzyme mediator of the adenosine pathway with other relevant clinical biomarkers such as PD-L1, immune infiltrates, and tumor mutation burden remains unknown. We surmised that understanding the contextual expression patterns of CD73 in LUAD can help us better understand the role of the adenosine pathway in NSCLC and in the immune pathobiology of this malignancy. Here, we sought to characterize the immunohistochemical expression of CD73 in a richly annotated cohort of early stage LUADs in association with various clinicopathological, molecular, immune features, and other markers involved in adenosine generation. We demonstrate that the extent of CD73 expression in malignant cells (MCs) defines groups of LUADs with distinct immune profiles and that thus may guide future personalized immunotherapeutic strategies.

## Materials and methods

### Patient samples

We first interrogated CD73 RNA expression in a set of 83 FFPE specimens from 50 patients representing different lesions in the sequence of LUAD pathogenesis including normal-appearing lung tissue (*n* = 38), adenomatous atypical hyperplasia (AAH; *n* = 9), adenocarcinoma in situ (AIS; *n* = 11), minimally invasive adenocarcinoma (MIA; *n* = 21), as well as invasive adenocarcinoma (*n* = 4), and that were profiled using the nCounter, PanCancer Immune Profiling Panel (NanoString Technologies) (Supplementary Fig. 1) in the manner described previously [23]. To determine associations of CD73 and LUAD clinicopathological, molecular, and immune features, we studied a cohort of LUADs (*n* = 106) from patients with early stage (stages I-III) disease that underwent surgical treatment without neoadjuvant therapy between Feb 1999 and 2012 at The University of Texas MD Anderson Cancer Center (MD Anderson; Houston, TX, USA). This study was approved by the MD Anderson Institutional Review Board and was conducted according to the principles of the Helsinki Declaration. Formalin-fixed paraffin-embedded (FFPE) LUADs tissue was placed in a tissue microarray (TMA); the tumor samples were selected based on the availability of FFPE tissue blocks; three 1 mm-diameter cores that included tissue from the center, intermediate, and peripheral areas of the tumor were used for the TMA, as previously described [24]. Detailed clinicopathological information, including demographics, smoking history, pathologic tumor-node-metastasis stage (staging system from the 8th American Joint Committee on Cancer) [25], histological patterns, and overall and recurrence-free survival were available for all cases and are summarized in Table 1. Briefly, the median age in this cohort was 65 years (range 41—84), with ever smokers representing 86% of patients included, and with a median follow-up of 86 months. Histological growth patterns were categorized as any-solid and non-solid based on the presence of any observed solid growth pattern found [26]. Mutational status of key driver genes, including KRAS, EGFR, STK11, TP53, and mutation burden derived from whole-exome sequencing [27] or Sanger sequencing data, were available in a subset of the cases (Table 1). Also, in a subset of this cohort (*n* = 65), next-generation sequencing RNA-based data using HTG EdgeSeq Precision Immuno-Oncology panel were employed to examine associations between CD73, CD38, and CD39 expression in LUAD and immune gene expression signatures [28–34] (SupplementaryTable 1), CD73 gene expression and its protein product by IHC.

**Table 1:** Clinicopathological and molecular characteristics of LUAD patients studied (*N* = 106)

Characteristic	*N* (%)
Age	
Median (range)	65 (41–84)
Sex	
Female	52 (49%)
Male	54 (51%)
Smoking history	
Never	15 (14%)
Current/former	91 (86%)
TNM 8th edition	
I	58 (55%)
II	26 (25%)
III	22 (20%)
Pathological T (8th)	
pT1a—pT2a	70 (66%)
pT2b—T4	36 (34%)
Pathological N (8th)	
N0	78 (74%)
N1	20 (19%)
N2	8 (7%)
Histologic pattern	
Any-solid	46 (43%)
Non-solid	60 (57%)
Molecular characteristics	
* EGFR* mutated	15 (15%)
* EGFR* wild type	85 (85%)
* KRAS* mutated	26 (25%)
* KRAS* wild type	77 (75%)
* TP53* mutated	27 (43%)
* TP53* wild type	36 (57%)
* STK11* mutated	7 (11%)
* STK11* wild type	56 (89%)
Mutation burden (number of mutations)	
Median (range)	145 (2–993)
Overall survival (median)	108.9 months
Death	59
Alive	47
Recurrence-free survival (median)	117.2 months
Recurrence	49
No recurrence	57

### Immunohistochemistry staining

We performed immune histochemistry (IHC) to detect the protein expression of CD73 (D7F9A), and CD39 (EPR20461). Antibody optimization of CD73 and CD39 was performed using tonsil tissue as control and multiple tumor specimens (including non-small cell lung carcinoma among others) to reach an optimal signal to noise ratio that can permit specific evaluation of cellular and subcellular expression patterns. Validation of the IHC assay included evaluation of FFPE lung cancer cell line pellets available with known mRNA expression of *CD73* and *CD39* (Supplementary Fig. 2). PD-L1 (E1L3N) and CD38 (SPC32) antibody immunohistochemistry validation, staining, and pathology evaluation were previously reported by our team [35]. The immunohistochemistry protocol is briefly described: tissue sections (4 μm) were stained in a Leica Bond Max automated stainer (Leica Biosystems Nussloch GmbH). The tissue sections were deparaffinized and rehydrated following the Leica Bond protocol. Antigen retrieval was performed for 20 min with Bond Solution #2 (Leica Biosystems, equivalent EDTA, pH 9.0) or Bond Solution # 1 (Leica Biosystems, equivalent Citrate Buffer, pH6). Primary antibodies were incubated for 15 min at room temperature and detected using the Bond Polymer Refine Detection kit (Leica Biosystems) with DAB as chromogen. The slides were counterstained with hematoxylin, dehydrated, and cover-slipped. Antibody clones and their vendor information as well as dilution and antigen retrieval conditions are summarized in SupplementaryTable 2.

### Immunohistochemistry scoring

**Fig. 1:**
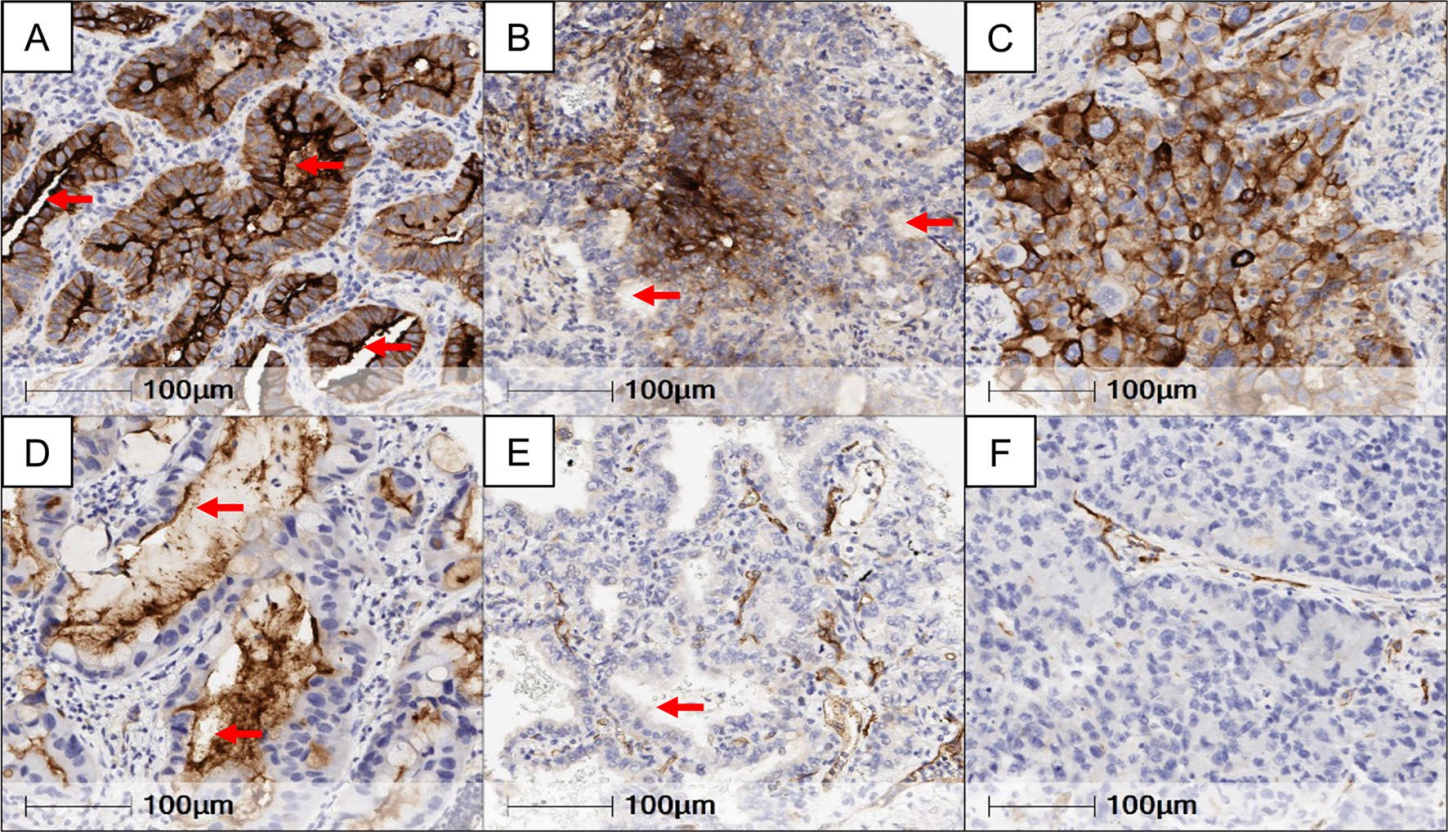
Immunohistochemical expression and localization of CD73 in resectable lung adenocarcinoma. Representative microphotographs showing different patterns of CD73 expression in the luminal and/or basolateral membrane of LUAD. **a** Luminal and basolateral membrane expression. **b** Basolateral membrane expression and with no immunoreactivity in the luminal compartment. **c** Basolateral membrane expression, with the absence of a lumen for evaluation. **d** Luminal membrane expression and with no immunoreactivity in the basolateral compartment. **e** Absence of expression in both the luminal and basolateral membranes. **f** No expression in the basolateral membrane and absence of a lumen. Red arrows indicate luminal membranes

Immunohistochemistry expression levels of CD73, was evaluated in malignant cells (MCs) by two pathologists (RS and LS), using standard microscopy. The percentage of MCs with any membrane CD73 expression was estimated. Basolateral (BL) (cell membrane not adjacent to luminal spaces) and luminal (L) membrane (cell membrane facing luminal spaces) expression levels of CD73 were separately scored when evaluable (Fig. 1). To determine total CD73 (T) expression in MCs, the average of BL and L scores was computed. Tumors were categorized as CD73 positive (T + , BL + or L + ,) based on the presence of any membrane expression in  > 1% of MCs. LUADs were stratified into three groups based on the extent of CD73 expression: ‘T Negative tumors’ (TN),  ≤ 1%, *n* = 27; ‘T Low group’ (TL),  < 55%  > 1%, *n* = 53, and ‘T High group’ (TH),  ≥  to 55%, *n* = 26). Lower quartile of CD73 percentage in malignant cells was used as cutoff for T Negative group, while the upper quartile was used as cutoff for T High group (Supplementary Fig. 3a).

Membrane or cytoplasmic CD39 expression in malignant cells was evaluated by two pathologists (DL and LS); since expression in malignant cells was not observed in any sample (0/95), expression levels of CD39 were evaluated in tumor stromal cells using digital image analysis supervised by a pathologist (DL). Briefly, the IHC-stained TMA slides were scanned using Aperio AT2 scanner (Leica Biosystem) at 20x. The digital images were visualized and analyzed with the HALO (IndicaLabs) software. A pathologist selected tumor stroma areas in each TMA core and applied algorithms to detect positive cells with cytoplasm or membrane expression of these markers; the results were expressed as cell densities (n/mm2) of the whole tumor stroma area analyzed; necrosis and artifacts were not included in the analysis.

Membrane PD-L1 was evaluated by two pathologists (DL and LS) as percentage of MCs with positive expression based on the International Association for the Study of Lung Cancer (IASLC) guidelines [36]. CD38 IHC expression annotated data included the evaluation in MCs and in tumor stromal cells, and were previously published by our team [35].

### Digital image analysis of tumor-associated immune cells

Immunohistochemistry and digital image analysis previously performed for a subset of LUADs (*n* = 94), included the analysis of cell densities of tumor-associated immune cells (TAICs): CD3 + (T cells), CD4 + (helper T cell), CD8 + (cytotoxic T cell), CD57 + (NK cells), granzyme B + (NK/cytotoxic T cells), CD45RO + (memory T cell), PD-1 + , FOXP3 + (regulatory T cell), and CD68 + (tumor-associated macrophages). The IHC methodology and image data analysis were performed as previously reported by our group [37, 38].

### Statistical analysis

CD73 mRNA expression across normal, preneoplastic, and malignant issues in the sequence of LUAD development was statistically determined using ANOVA and Benjamini–Hochberg correction. Targeted immune gene expression data were first median-normalized and then log2 transformed for further analysis. Scores of previously curated immune gene signatures were calculated by computing average expression of genes within each signature. To determine the association of categorical CD73 expression (T + , BL +, and L + , and T high, T Low, and T negative) with clinicopathological characteristics, we used Fisher’s exact test, as appropriate for categorical data. To test association between continuous and categorical variables, Wilcoxon signed-rank test and Kruskal–Wallis were applied for categorical variables with two levels or more than two levels, respectively. To correlate the association between continuous CD73 expression, immune markers, and immune signatures, we used Spearman’s rank correlation, and scatterplots. For survival analysis, we used Cox proportional-hazard model with CD73 expression as continuous and categorical variables separately. Heat maps of CD73 expression and tumor-associated immune cells and PD-L1 expression were generated after normalizing values for better visualization of data.

## Results

### Membrane expression patterns of CD73 and their association with clinicopathological features and immune biomarkers

We first interrogated expression patterns of CD73 in the pathogenesis of LUAD. We evaluated the expression of *CD73* mRNA in a series of premalignant lesions, along with malignant tumors, representing the sequence of pathogenesis of LUAD (83 specimens from 50 patients). We found that *CD73* expression was significantly and progressively increased across normal-appearing lung tissue, AAH, AIS, MIA, and adenocarcinoma (p < 0.0001; Supplementary Fig. 1). These findings prompted us to comprehensively examine CD73 protein expression patterns in a larger cohort of early stage LUAD.

**Fig. 2:**
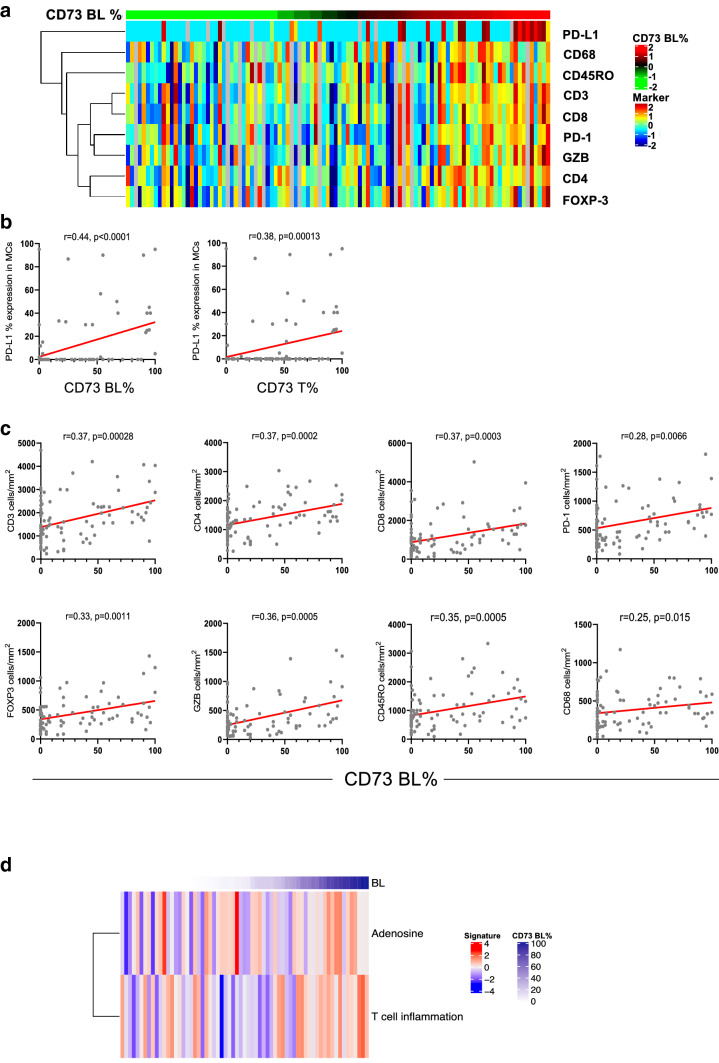
Basolateral CD73 expression is associated with higher immune infiltration in lung adenocarcinoma. **a** Heat map of TAIC densities and PD-L1 (% of expression) in MCs from 95 LUADs sorted according to BL CD73 expression (red, relatively higher BL CD73 expression; green, lower BL CD73 expression). Rows represent immune marker and columns denote samples (red, relatively higher TAIC density or PD-L1%; blue, lower TAIC density or PD-L1%). **b** Spearman correlation analysis of PD-L1 expression in MCs with BL and T CD73. **c** Spearman correlation analyses of TAICs (*y*-axis) with BL CD73 expression (*x*-axis)

In our cohort of resectable early stage LUAD, immunohistochemistry evaluation revealed a positive total (T +) CD73 expression (> 1%) in 75% (79/106) of LUADs. Positive basolateral (BL +) expression was found in 60% (68/106); positive luminal (L +) was present in 83% (60/72) of LUADs that had luminal structures in the TMA cores. Positive correlation was found between BL and L CD73 expression (*r* = 0.49 *p* = 0.0042). Detailed information on L and BL co-expression is presented in Supplementary Table 3. Associations of CD73 expression with clinicopathological characteristics are shown in Supplementary Table 4. In our cohort, never smokers showed higher rates of T + CD73 expression (15/15, 100%) compared to ever smoker patients (64/91, 70%) (*p* = 0.0107). Tumors with any-solid histological pattern were associated with lower frequencies of T + CD73 (*p* = 0.0243). LUADs from female patients had higher frequency of BL + CD73 (*p* = 0.0268). We did not find correlations between CD73 expression and survival outcomes (data not shown). T and BL expression levels showed positive correlation with most immune biomarkers evaluated by IHC (Fig. 2a and Supplementary Fig. 4a). Specifically, CD73 T and BL expression levels correlated with higher PD-L1 expression (*r* = 0.38, *p* = 0.0013 and, *r* = 0.44,* p* < 0.0001, respectively) (Fig. 2b) and with higher densities of CD3, CD4, CD8, CD45RO, CD68, PD-1, FOXP3, and Granzyme B positive cells (all *p* < 0.05; Fig. 2c, and Supplementary Fig. 4a). L CD73 levels positively correlated with CD3 and CD4 cell densities (Supplementary Fig. 4b).

In a subset of patients (*N* = 65), we performed RNA-sequencing-based analysis of immune genes and signatures using the HTG EdgeSeq platform. Corroborating our immunohistochemical analyses, BL CD73 gene expression was positively associated with increased expression of T-cell inflammation (*p* = 0.013) and adenosine signatures (*p* = 0.035) (Fig. 2d).

### CD73 expression defines subgroups of LUADs with disparate clinicopathological and immune features

**Fig. 3:**
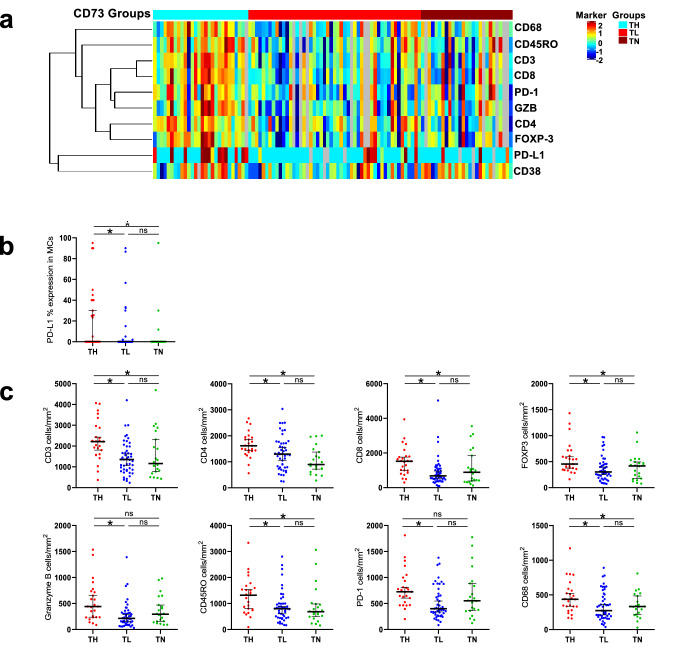
Extent of CD73 expression defines groups of lung adenocarcinoma with disparate tumor immune infiltration. **a** Heat map of TAIC densities and PD-L1 (% of expression) in MCs of 95 LUADs grouped based on the extent of CD73 expression (TH, TL, and TN). Rows represent immune markers and columns denote samples (red, relatively higher TAIC density or PD-L1%; blue, relatively lower TAIC density or PD-L1%). **b** Plots showing PD-L1% expression among the TH, TL, and TN groups. **c)** Plots showing TAIC densities among the TH, TL, and TN groups (*p < 0.05 based on the Kruskal–Wallis test, *n.s.* not significant, bars correspond to median values ± 95% CI)

We then defined groups of LUADs (designated as high, low, and negative) based on the extent of CD73 membrane expression. CD73 IHC expression across the three groups significantly and positively correlated with its RNA counterpart (*p* < 0.0001, Supplementary Fig. 3b). Based on the extent of CD73 expression, we stratified our cohort into three groups: ‘T Negative tumors’ (TN),  ≤ 1%, *n* = 27; ‘T Low group’ (TL),  > 1% and  < 55%, *n* = 53; and ‘T High group’ (TH),  ≥  to 55%, *n* = 26). L + and BL + expressions in these groups are shown in Supplementary Table 4. We found that these CD73-defined groups correlated with tobacco history (*p* = 0.0194); most LUADs from never smoker patients were TL (73%) and none of them were TN. TL LUADs showed more frequent non-solid histological patterns, while TH and TN showed similar proportions of tumors with any-solid histological pattern (*p* = 0.0003) (Table 2). Notably, the CD73-defined groups correlated with most of the immune markers examined (Fig. 3a). TH showed the highest PD-L1 expression in MCs (*p* = 0.002) (Fig. 3b), and significantly higher cell densities of CD3 CD4, CD8, PD-1, FOXP3, Granzyme B, CD45RO, and CD68-positive cells (Fig. 3c). In addition, 22.2% of tumors evaluated co-expressed CD73 and PD-L1. We did not find significant differences in immune marker expression between TN and TL LUADs. No differences in survival outcomes among the three groups were observed (Supplementary Fig. 5).

**Table 2:** Clinicopathological and molecular features of LUAD patients grouped based on extent of CD73 expression

Characteristic	*N*	T high (TH) (26/106, 25%)	T low (TL) (53/106, 50%)	T negative (TN) (27/106, 25%)	*p* values^a^
		*N* (%)	*N* (%)	*N* (%)	
Age					
≤ 65	53	17 (32%)	21 (40%)	15 (28%)	0.2069
> 65	53	11 (21%)	30 (57%)	12 (23%)	
Sex					
Female	52	15 (29%)	26 (50%)	11 (21%)	0.6116
Male	54	13 (24%)	25 (46%)	16 (30%)	
Smoking history					
Never	15	4 (27%)	11 (73%)	0 (0%)	0.0194
Current/former	91	24 (26%)	40 (44%)	27 (30%)	
TNM 8th edition					
I	58	18 (31%)	28 (48%)	12 (20%)	0.4244
II	26	4 (15%)	12 (46%)	10 (39%)	
III	22	6 (27%)	11 (50%)	5 (23%)	
Pathological T (8th)					
pT1a—pT2a	70	19 (27%)	35 (50%)	16 (23%)	0.6934
pT2b—T4	36	9 (25%)	16 (44%)	11 (31%)	
Pathological N (8th)					
N0	78	22 (28%)	37 (47%)	19 (24%)	0.9041
N1	20	5 (25%)	10 (50%)	5 (25%)	
N2	8	1 (13%)	4 (50%)	3 (37%)	
Histologic pattern					
Any-solid	46	15 (32%)	14 (30%)	17 (37%)	0.0003
Non-solid	60	11 (18%)	39 (65%)	10 (17%)	
Molecular features					
* EGFR* mutated	15	5 (33%)	9 (60%)	1 (7%)	0.1717
* EGFR* wild type	85	22 (26%)	38 (45%)	25 (29%)	
* KRAS* mutated	26	8 (31%)	13 (50%)	5 (19%)	0.7039
* KRAS* wild type	77	20 (26%)	36 (47%)	21 (27%)	
* TP53* mutated	27	11 (41%)	7 (26%)	9 (33%)	0.0035
* TP53* wild type	36	5 (14%)	24 (67%)	7 (19%)	
* STK11* mutated	7	1 (14%)	2 (29%)	4 (57%)	0.1901
* STK11* wild type	56	15 (27%)	29 (52%)	12 (21%)	
Mutational burden					
Median	63	353 (2–955)	154 (3–914)	392 (33–993)	0.0018

### Association of CD73 expression with molecular features

We then examined the association of CD73 expression with genomic features in LUAD. TH and TN groups showed significantly higher proportion of *TP53* mutant tumors (*p* = 0.0035) compared to TL group. Somatic mutation burdens were significantly lower in the TL group compared to TH and TN groups (*p* = 0.0018) (Table 2). We found that L negative CD73 LUADs comprised more *STK11* mutant LUADs rates compared to L +  (*p* = 0.0041), although in our cohort, we only have a small number of *STK11* mutations (*n* = 7). We did not find associations between *KRAS* and *EGFR* mutations with CD73 expression.

### Association of CD73 with other markers involved in adenosine generation

We next interrogated other critical enzymes in the adenosine pathway, namely CD38 and CD39. CD38 expression in malignant cells was found in 20% (20/98) of LUADs and 18 (89%) of them co-expressed T CD73. Assessment of CD38 in tumor stroma showed that higher number of CD38^+^ cells positively associated with the TN group (*p* = 0.02) (Supplementary Fig. 6a). In addition, our immune gene profiling analysis showed that CD38 cell densities in tumor stroma were associated with specific immune cell signatures indicative of T-cell inflammation, cytotoxic T lymphocytes, expanded host immune responses, tumor inflammation (TIS), interferon-gamma signaling, as well as peripheral T-cell infiltration and M1 macrophage polarization (Supplementary Fig. 6b). In contrast, CD39 expression was not observed in malignant cells in our cohort, and CD39^+^ cell densities in the tumor stroma did not exhibit significant association with CD73 expression (Supplementary Fig. 6c) or with immune signatures obtained by gene expression analysis.

## Discussion

The immunosuppressive adenosine pathway, in which CD73 plays a critical role, has been proposed as one of the possible mechanisms of resistance to ICI [9, 35, 39]. The current combination of ICI therapies and anti-CD73 antibodies are attractive therapeutic approaches, and are under evaluation [40] with the aim to improve the outcome in patients with NSCLC that did not response to ICI (monotherapy or combination with chemotherapy). Yet, the role of CD73 in the pathobiology and immune contexture of LUAD is poorly understood. To begin to fill this void, we examined the expression patterns of CD73 in a richly annotated cohort of early stage LUADs in association with various features including clinicopathological, molecular, and immune covariates. We found that CD73 was expressed in a significant fraction (75%) of LUADs and categorized subsets of LUAD with distinct histological, molecular, and immune features.

In contrast to previous studies that mostly focused on total CD73 expression assessment, we interrogated CD73 in different membrane compartments (basolateral and luminal; BL and L, respectively) of MCs. Our pathological analyses demonstrated that tumors with different CD73 expression patterns exhibited distinct clinicopathological (e.g., histological patterns) and molecular associations, possibly pointing to causal links between CD73 expression or membrane localization and tumor differentiation [9, 41]. This hypothesis is also supported by our finding on progressively increased expression of *CD73* across premalignant lung lesions representing different stages in the sequence of LUAD pathogenesis. We also found that the localization of CD73 in cells from well-differentiated LUADs was predominantly luminal, which may as well be related to the physiological protective and mitigating properties of CD73 against inflammation (45). Of note, we found distinct associations between not only the presence or absence of CD73 but also the extent of expression of this antigen with smoking status, molecular features, and immune infiltration (Table 1). Consistent with previous reports [22], our cohort showed that all (100%) never smoker LUADs exhibited positive CD73 expression when compared to smoker tumors (70%). However, among positive CD73 tumors, never smoker patients had lower extent of CD73 expression (T Low group), along with more differentiated tumors, less mutation burden, and lower rates of p53 mutation. These results suggest that extensive expression of CD73 in tumors from smoker patients could be in part explained by the higher immune infiltration observed in these tumors. Interestingly, CD73 membrane localization was also predominantly luminal (Supplementary Table 5), while the group with higher extent of CD73 expression, the predominant localization was basolateral. It is reasonable to surmise that CD73, viz., its disparate localization, may have distinct roles in the molecular pathogenesis of smoker and non-smoker LUADs. It is also plausible to suggest that CD73 membrane localization may have important implications on the effectiveness of anti-CD73 therapy.

It is important to mention that we observed that tumor BL CD73 expression positively correlated with features of a “hot” immune environment such as PD-L1 and immune cell infiltration rendering the plausible supposition that CD73 immune function may be disparate between BL and L compartments of LUAD cells. Similarly, when we analyzed immune cell densities within LUADs grouped based on CD73 positivity, the TH group displayed elevated PD-L1 and immune cell infiltration compared with the TL and TN groups. It is noteworthy, that CD73 was shown to suppress anti-tumor immunity and promote immune evasion [9, 42, 43]. Thus, given our findings along with the previous reports on CD73 function, it is not unreasonable to suggest that expression of CD73 may underlie inferior response to ICI even in tumors with concomitant high tumoral PD-L1 expression and immune cell infiltration [44, 45]. In line with our results, a previous report demonstrated that high levels of adenosine correlated with elevated infiltration of immune cells, but with a decreased response to ICI [32]. It is intriguing to suppose that targeting CD73 may enhance anti-tumor immunity, particularly in tumors with high levels of CD73, as well as augment the effect of ICI. Indeed, targeting CD73 was shown to skew the immune TME to a more anti-tumor phenotype in preclinical models [46, 47]. In separate context, our findings also suggest that targeting CD73 may help augment anti-tumor immunity in LUADs with low yet positive CD73, and which we find here to exhibit a relatively “cold” immune contexture [48]. Of note, we found that a fraction of LUADs that were CD73 negative displayed abundant expression of CD38 concomitant with a muted host immune response, suggesting redundant activation of the non-canonical adenosine pathway [9, 11] in these tumors and their potential tractability by agents that target this pathway. Our study points to the potential role of CD73, and other members of the adenosine signaling pathway, as potential mechanisms of tumor immune evasion and resistance to ICI, thus providing additional rationale for propagating anti-CD73 antibodies in new combinatorial immunotherapeutic regimens. As mentioned before, we found that differential (e.g., BL vs. L) CD73 localization was associated with distinct clinicopathological and molecular features in LUAD. It is intriguing to propose that in-depth assessment of CD73 expression along with its membrane localization will provide comprehensive assessment of patients who may benefit from agents targeting this immune marker.

Our study is not without limitations. It is important to mention that we interrogated tissue microarrays of LUAD, with these arrays typically harboring relatively small tissue cores which may bring about increased tumor and, thus, immune marker heterogeneity and under-representation of luminal structures of adenocarcinomas—thus warranting future studies probing CD73 in whole tissue specimens. It is also noteworthy, given our study design and goals, that our cohort was primarily comprised of resectable early stage tumors with, thus, under-representation of relatively more advanced (e.g., metastatic) LUADs. In this context, our study is unable to ascertain relative patterns of CD73 expression (and localization), along with features of host anti-tumor immunity and immune evasion, between early stage and more advanced LUADs. Since mechanisms of host immune evasion by the tumor, along with genomic and mutational complexity, are expectantly more pronounced in advanced-stage tumors, future studies are warranted to fully probe CD73, and other members of the adenosine pathway, along the continuum of different stages (e.g., early, local/oligometastatic to distant metastatic) in LUAD. Furthermore, the expression patterns of CD73 in patients who have received ICI, preoperatively or in the advanced setting, are not yet discerned. It also cannot be neglected that our study is retrospective in nature and comprises a cohort of limited number of patients and from a single center warranting validation of our findings by external cohorts. Additionally, future studies are warranted that further probe mechanisms involving CD73 expression and its interaction with host immune responses in LUAD. It is important to note that, unlike earlier work [22], we did not find associations between CD73 with clinical outcome and *EGFR* mutation status. This discrepancy may be attributable to the disparate patient molecular and clinicopathological profiles in our cohort compared to those earlier studies that focused on East Asian patients [22]. Due to the lack of tissue availability, the analysis of *CD73* gene expression in AAH, MIA, AIS, LUAD, and normal lung tissue was not validated by IHC. Nonetheless, our study provides new and comprehensive insights into diverse patterns of CD73 expression and localization, in association with genomic, immune, and clinical features, in early stage LUAD, thus offering a roadmap in the future to interrogate the role of CD73 expression in immunotherapy and/or response to ICI.

In conclusion, we comprehensively surveyed the expression, abundance, and membrane tumor localization of CD73 in early stage LUAD, and found that this immune marker with distinct clinicopathological, molecular, and immune characteristics. Our findings on increased expression of the immune evasion mediator CD73 in LUADs with elevated PD-L1 and immune cell infiltration offer potential insight into why some patients with augmented immune response still respond poorly or modestly to ICI. Our data also provide the plausible rationale for exploring immunotherapeutic regimens consisting of anti-CD73 antibodies in combination with ICI.

### Supplementary Information

Below is the link to the electronic supplementary material.

Supplementary file1 Supplementary Fig.1 Increased CD73 gene expression in the pathogenesis of LUAD. CD73 expression was examined in normal lung tissues, atypical adenomatous hyperplasia (AAH), adenocarcinoma in situ (AIS), minimally invasive adenocarcinoma (MIA), and in adenocarcinoma (ADC) as described in the Materials and Methods section. Differences in CD73 expression (log base 2 transformed) among all groups was determined using ANOVA and plotted. Supplementary Fig.2 Optimization and validation of CD73 and CD39 immunohistochemistry in tonsil tissue and cell lines. CD73 IHC staining in reactive tonsil tissue a was observed mainly in the membrane and cytoplasm of immune cells from germinal centers, mantle zone, interfollicular spaces, basal layer of reticulated epithelium, fibroblasts, endothelial cells, and in scattered intraepithelial immune cells. CD39 IHC staining in tonsil tissue b was observed in the membrane and cytoplasm of scattered immune cells from germinal center, interfollicular areas and in intraepithelial immune cells. CD73 levels of IHC staining in cell lines c was negative in: NCIH82, NCIH1694, NCIH1341 and NCIH522; and positive in: NCIH196, NCIH596, HCC4006, NCIH1838 and NCIH650, concordant with mRNA data. CD39 IHC staining was negative in all cell lines d, concordant with negative or low levels of mRNA of CD39 expression. e Table showing histopathology evaluation of membrane IHC expression of CD73 and CD39 from different cell lines c, d. IHC microphotographs, 20x. Scale bar 100μm. *Gene expression values extracted from CCLE (Cancer cell line encyclopedia). (CD73 (NT5E):https://portals.broadinstitute.org/ccle/page?gene=NT5E; CD39 (ENTPD1): https://portals.broadinstitute.org/ccle/page?gene=ENTPD1). Supplementary Fig.3 Grouping of lung adenocarcinomas based on extent of CD73 expression. a Distribution of IHC T CD73 (%) expression among the 106 LUADs. b Plots showing CD73 mRNA expression levels among the predefined CD73 groups (TH, TL and TN). (*p<0.05 based on the Kruskal-Wallis test, n.s. not significant, bars correspond to median values ± 95% CI). Supplementary Fig.4 Total and luminal CD73 is associated with augmented tumor immune infiltration in lung adenocarcinoma. Spearman correlation analyses of TAIC densities with total a and luminal b CD73 expression by IHC. Supplementary Fig.5 Survival analysis. Analysis for Recurrence-free survival (a), and Overall survival (b) in CD73 groups (TH, TL and TN). Supplementary Fig.6 Association of CD73 expression with other markers involved in adenosine generation. Correlation of CD73 expression in malignant cells with a) CD38+ cell densities in tumor stroma, b Heatmap of immune gene signatures from 65 LUADs sorted according to CD38+ cell densities in tumor stroma (purple, relatively higher CD38+cell densities; white, lower CD38+ cell densities) c Correlation of CD73 expression in malignant cells with CD39+ cell densities (*p<0.05 based on the Kruskal-Wallis test, n.s. not significant, bars correspond to median values ± 95% CI).(PDF 1459 KB)

Supplementary file2 (PDF 62 KB)

## Data Availability

Data are available upon reasonable request. The data that support the findings of our study are available upon request from the corresponding author. The data are not publicly available due to privacy or ethical restrictions.

## Abbreviations

NSCLC: Non-small cell lung cancer
LUADs: Lung adenocarcinomas
T: Total
BL: Basolateral
L: Luminal
TH: Total high group
TL: Total low group
TN: Total negative group
TAICs: Tumor-associated immune cells.
ICI: Immune checkpoint inhibitors
PD-1: Programmed death 1
PD-L1: Programmed death-Ligand 1
CTLA-4: Cytotoxic T lymphocyte-associated 4
NK: Natural killer
TME: Tumor microenvironment
FFPE: Formalin-fixed paraffin-embedded
MCs: Malignant cells
