# Correction to: CD73 expression defines immune, molecular, and clinicopathological subgroups of lung adenocarcinoma

**DOI:** 10.1007/s00262-021-02893-9

**Published:** 2021-03-09

**Authors:** Pedro Rocha, Ruth Salazar, Jiexin Zhang, Debora Ledesma, Jose L. Solorzano, Barbara Mino, Pamela Villalobos, Hitoshi Dejima, Dzifa Y. Douse, Lixia Diao, Kyle Gregory Mitchell, Xiuning Le, Jianjun Zhang, Annikka Weissferdt, Edwin Parra-Cuentas, Tina Cascone, David C. Rice, Boris Sepesi, Neda Kalhor, Cesar Moran, Ara Vaporciyan, John Heymach, Don L. Gibbons, J. Jack Lee, Humam Kadara, Ignacio Wistuba, Carmen Behrens, Luisa Maren Solis

**Affiliations:** 1 Department of Translational Molecular Pathology, The University of Texas MD Anderson Cancer Center, 2130 West Holcombe Boulevard, Houston, TX 77030 USA; 2 Universidad de Barcelona, Barcelona, Spain; 3 Department of Bioinformatics and Comp Biology, The University of Texas MD Anderson Cancer Center, Houston, TX USA; 4 Thoracic/Head and Neck Medical Oncology, The University of Texas MD Anderson Cancer Center, Houston, TX USA; 5 Thoracic and Cardiovascular Surgery, The University of Texas MD Anderson Cancer Center, Houston, TX USA; 6 The University of Texas MD Anderson Cancer Center, Houston, TX USA

## Correction to: Cancer Immunology, Immunotherapy https://doi.org/10.1007/s00262-020-02820-4

The original version of this article unfortunately contained a mistake. The presentation of Fig. 2 was incorrect.

The corrected Fig. 2 is given in following page.

**Fig. 2:**
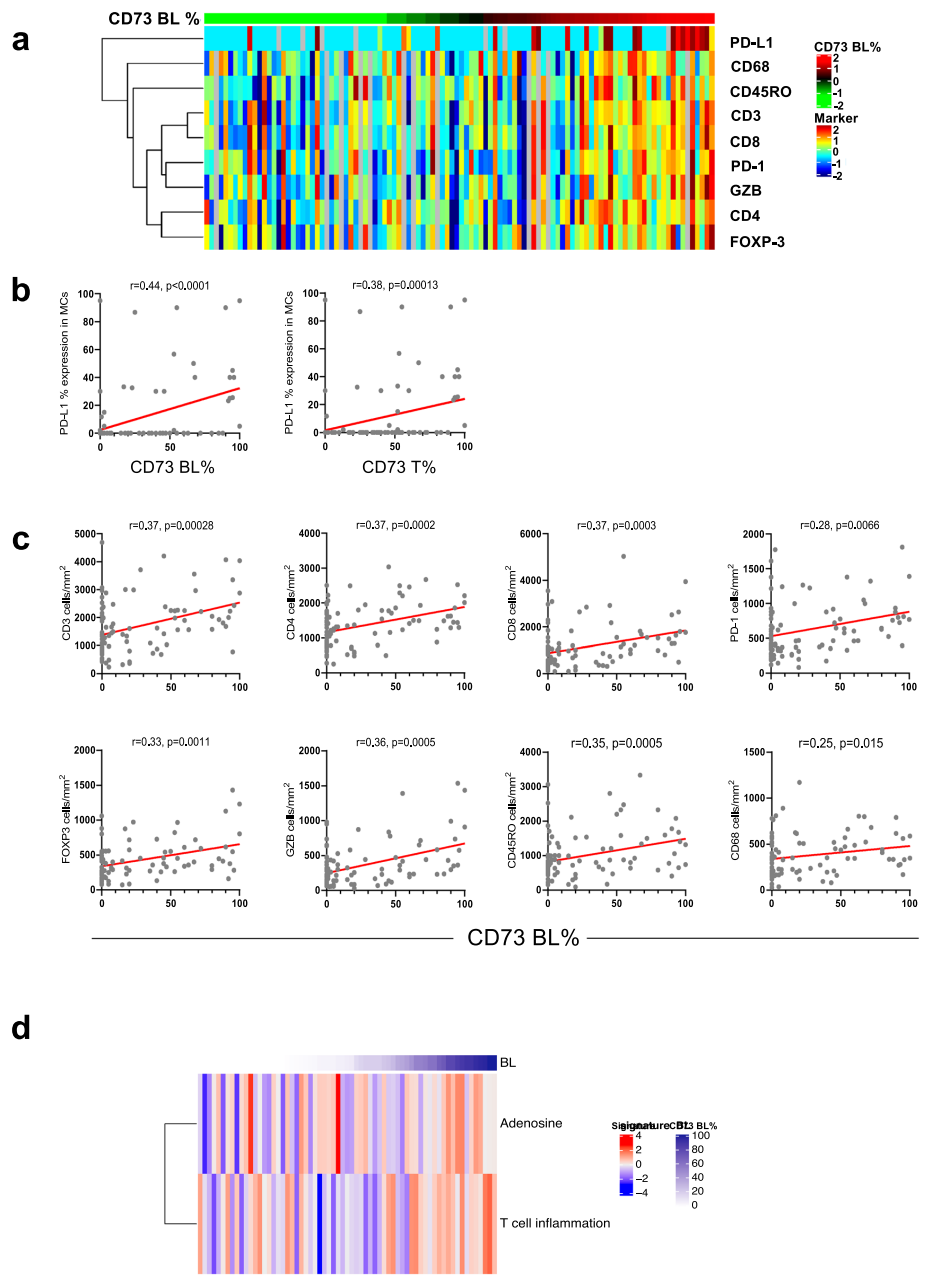
Basolateral CD73 expression is associated with higher immune infiltration in lung adenocarcinoma. **a** Heat map of TAIC densities and PD-L1 (% of expression) in MCs from 95 LUADs sorted according to BL CD73 expression (red, relatively higher BL CD73 expression; green, lower BL CD73 expression). Rows represent immune marker and columns denote samples (red, relatively higher TAIC density or PD-L1%; blue, lower TAIC density or PD-L1%). **b** Spearman correlation analysis of PD-L1 expression in MCs with BL and T CD73. **c** Spearman correlation analyses of TAICs (*y*-axis) with BL CD73 expression (*x*-axis)

The original article has been corrected.

