# Supplementary material for: CD73 expression defines immune, molecular, and clinicopathological subgroups of lung adenocarcinoma
Source: Cancer Immunol Immunother. 2021 Jan 8;70(7):1965–76. doi: 10.1007/s00262-020-02820-4 (PMC8195808; doi:10.1007/s00262-020-02820-4)

Supplementary Figure 1

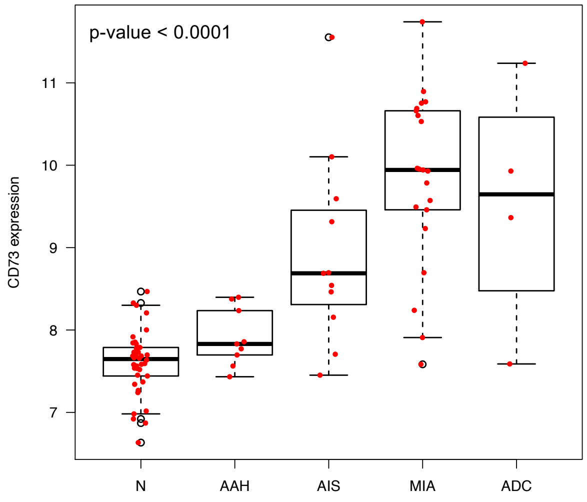

Supplementary Figure 2

a

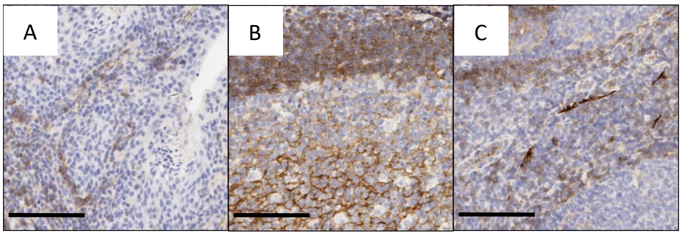

b

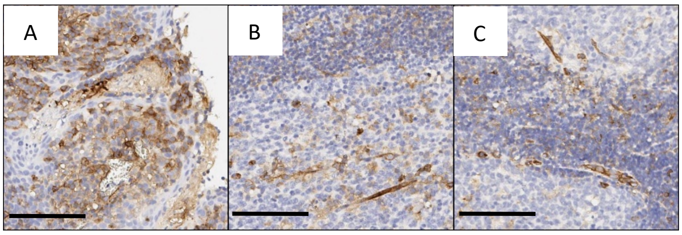

c

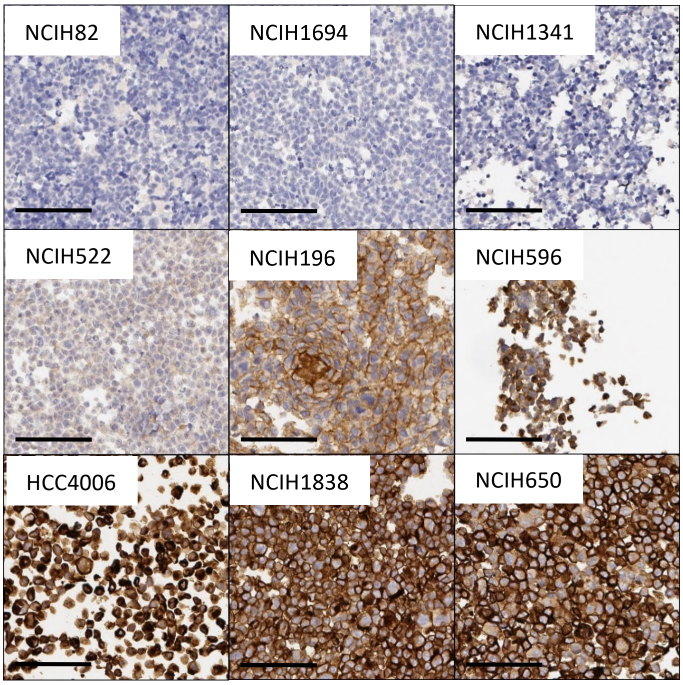

d

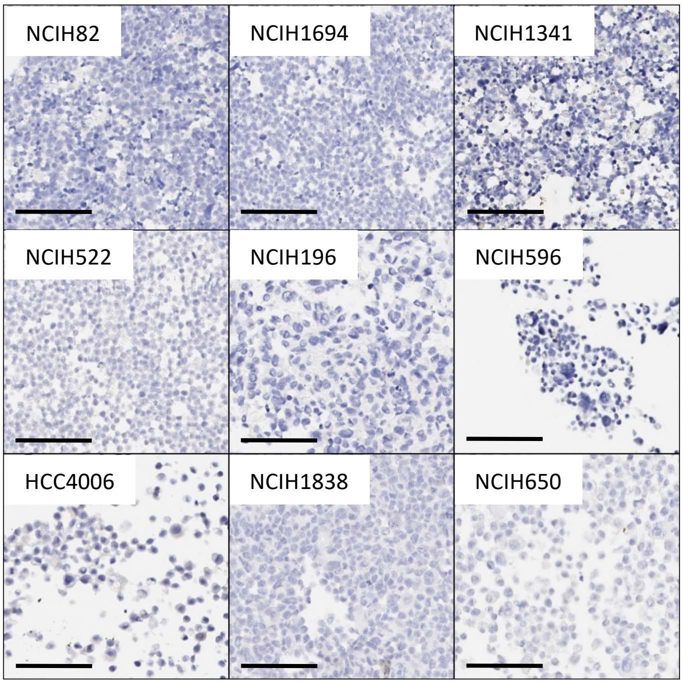

e

Cell line	*CD73* mRNA*	CD73 IHC Membrane	*CD39* mRNA*	CD39 IHC Membrane
NCIH82	-4.51793	0	2.379744	0
NCIH1694	-3.78588	0	2.788662	0
NCIH1341	-1.9469	0	2.226699	0
NCIH522	-1.16531	0	2.266061	0
NCIH196	3.111517	2+	-0.20666	0
NCIH596	4.066031	2+	-0.55199	0
HCC4006	5.828985	3+	-1.03664	0
NCIH1838	7.296265	3+	1.27548	0
NCIH650	7.760806	3+	1.163237	0

Supplementary Figure 3

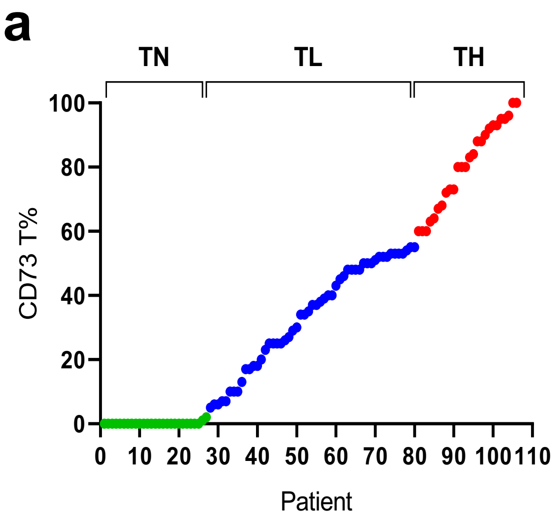

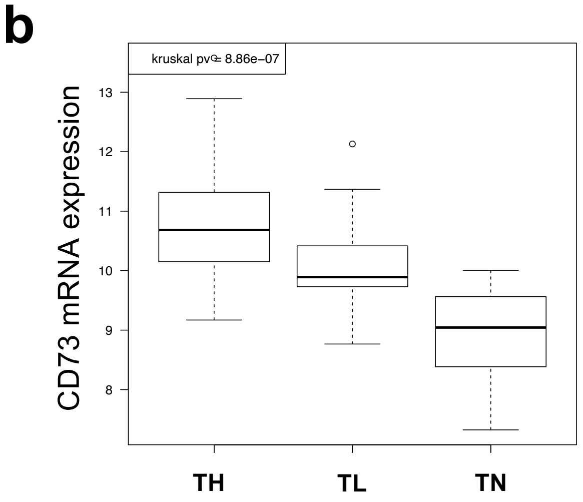

Supplementary Figure 4

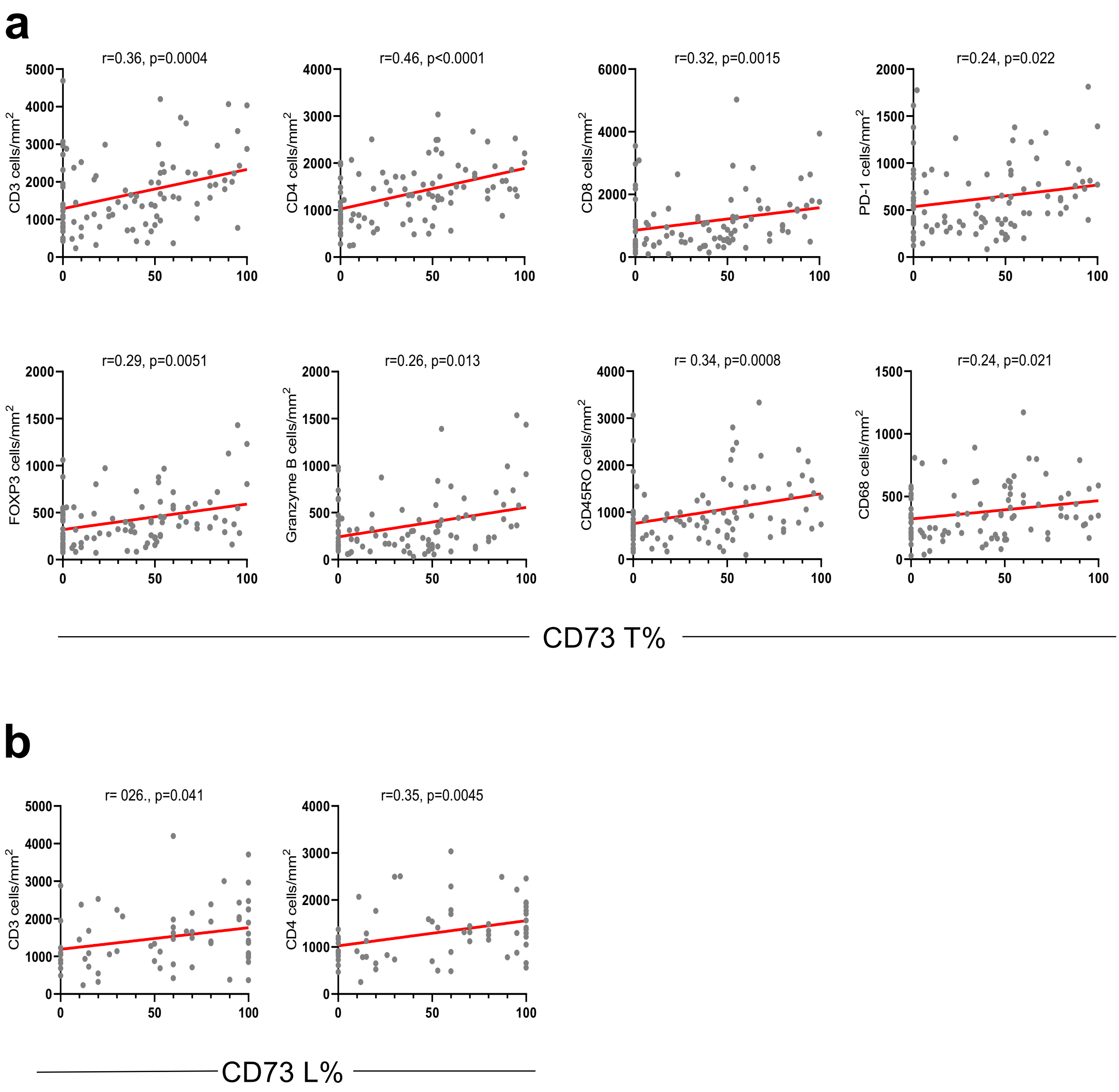

Supplementary Figure 5

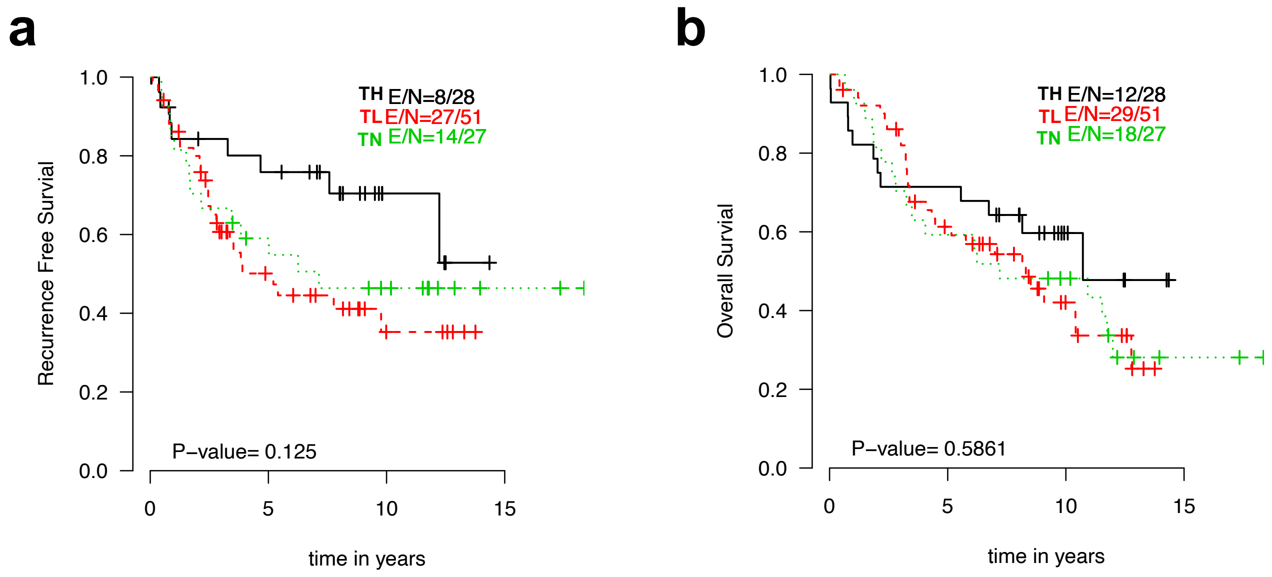

Supplementary Figure 6

a

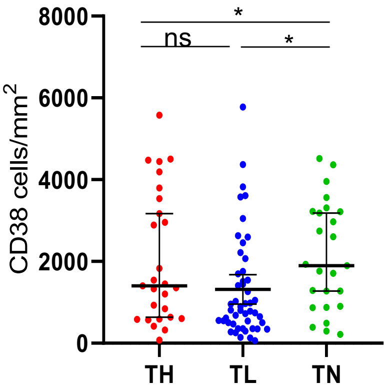

b

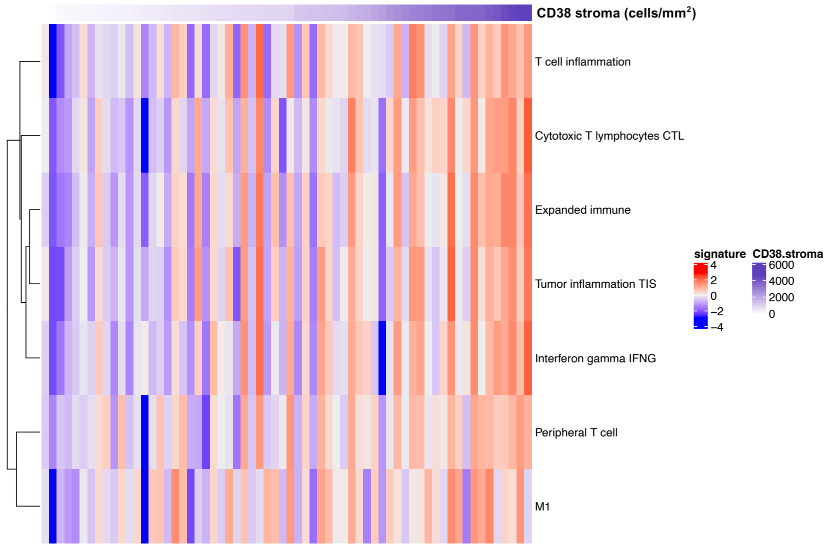

c

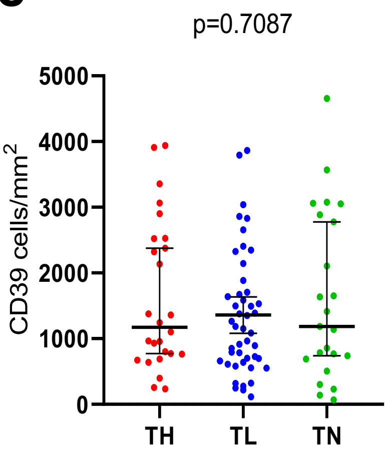

Supplement: Supplementary file 1 — Supplementary file1 Supplementary Fig.1 Increased CD73 gene expression in the pathogenesis of LUAD. CD73 expression was examined in normal lung tissues, atypical adenomatous hyperplasia (AAH), adenocarcinoma in situ (AIS), minimally invasive adenocarcinoma (MIA), and in adenocarcinoma (ADC) as described in the Materials and Methods section. Differences in CD73 expression (log base 2 transformed) among all groups was determined using ANOVA and plotted. Supplementary Fig.2 Optimization and validation of CD73 and CD39 immunohistochemistry in tonsil tissue and cell lines. CD73 IHC staining in reactive tonsil tissue a was observed mainly in the membrane and cytoplasm of immune cells from germinal centers, mantle zone, interfollicular spaces, basal layer of reticulated epithelium, fibroblasts, endothelial cells, and in scattered intraepithelial immune cells. CD39 IHC staining in tonsil tissue b was observed in the membrane and cytoplasm of scattered immune cells from germinal center, interfollicular areas and in intraepithelial immune cells. CD73 levels of IHC staining in cell lines c was negative in: NCIH82, NCIH1694, NCIH1341 and NCIH522; and positive in: NCIH196, NCIH596, HCC4006, NCIH1838 and NCIH650, concordant with mRNA data. CD39 IHC staining was negative in all cell lines d, concordant with negative or low levels of mRNA of CD39 expression. e Table showing histopathology evaluation of membrane IHC expression of CD73 and CD39 from different cell lines c, d. IHC microphotographs, 20x. Scale bar 100μm. *Gene expression values extracted from CCLE (Cancer cell line encyclopedia). (CD73 (NT5E):https://portals.broadinstitute.org/ccle/page?gene=NT5E; CD39 (ENTPD1): https://portals.broadinstitute.org/ccle/page?gene=ENTPD1). Supplementary Fig.3 Grouping of lung adenocarcinomas based on extent of CD73 expression. a Distribution of IHC T CD73 (%) expression among the 106 LUADs. b Plots showing CD73 mRNA expression levels among the predefined CD73 groups (TH, TL and TN). (*p<0 [file 262_2020_2820_MOESM1_ESM.pdf]
