# Supplementary material for: CD73 expression defines immune, molecular, and clinicopathological subgroups of lung adenocarcinoma
Source: Cancer Immunol Immunother. 2021 Jan 8;70(7):1965–76. doi: 10.1007/s00262-020-02820-4 (PMC8195808; doi:10.1007/s00262-020-02820-4)
Supplement: Supplementary file 2 — Supplementary file2 (PDF 62 KB) [file 262_2020_2820_MOESM2_ESM.pdf]

SUPPLEMENTARY TABLES.

Supplementary table 1. Annotated genes included to define the immune gene signatures.

Signature	Reference	Genes included
Adenosine	Sidders et al.[32]	PPARG,CYBB,COL3A1,FOXP3,LAG3,APP,GPI,PTGS2,CASP1,FOS,MAPK1,MAPK3
T cell inflammation	Spranger et al.[29]	CD8A,CCL2,CCL3,CCL4,CXCL9,CXCL10,ICOS,GZMK,IRF1,HLA-DMA,HLA-DMB,HLA-DOA,HLA-DOB
Cytotoxic T lymphocytes (CTL)	Jian et al.[30]	CD8A,CD8B,GZMA,GZMB,PRF1
Expanded immune	Ayers et al.[28]	CD3D,IDO1,CIITA,CD3E,CCL5,GZMK,CD2,CXCL13,NKG7,HLA-E,CXCR6,LAG3,TAGAP,CXCL10,STAT1,GZMB
Tumor inflammation (TIS)	Danaher et al.[33]	PSMB10,HLA-DQA1,HLA-DRB1,HLA-E,NKG7,CD8A,CCL5,CXCL9,CD27,CXCR6,IDO1,STAT1,TIGIT,LAG3,CD274
Interferon-gamma (IFNG)	Ayers et al.[28]	IFNG,STAT1,CCR5,CXCL9,CXCL10,CXCL11,IDO1,PRF1,GZMA,HLA-DRA
Peripheral T cell	Hwang et al.[31]	HLA-DOA,GPR18,STAT1
M1	Hwang et al.[31]	CCR7,CD27,CD48,FOXO1,HLA-B,HLA-G,IFIH1,IKZF4,LAMP3,NFKBIA,SAMHD1

Supplementary table 2. Information of antibodies used for immunohistochemistry analysis

Biomarker	Clone	Vendor	Catalogue #	Antigen Retrieval	Dilution
PD-L1	E1L3N	Cell Signaling	13684	Epitope Retrieval #1 (Citrate Buffer ph6)	1:100
CD38	SPC32	Leica/Novocastra	NCL-L-CD38-290	Epitope Retrieval #1 (Citrate Buffer ph6)	1:100
CD39	EPR20461	Abcam	ab223843	Epitope Retrieval #1 (Citrate Buffer ph6)	1:500
CD73	D7F9A	Cell Signaling	13160S	Epitope Retrieval #2 (Tris-EDTA Buffer)	1:200

Supplementary table 3. Overview of Luminal (L) and Basolateral (BL) membrane expression of CD73 in LUADs

Basolateral CD73 expression	Luminal CD73 expression			Total
	L CD73+	L CD73-	Non-lumen	
	N (%)	N (%)	N (%)	N (%)
BL CD73+	44 (42)	1 (1)	19 (18)	64 (60)
BL CD73-	16 (15)	11 (10)	15 (14)	42 (40)
Total	60 (57)	12 (11)	34 (32)	106 (100)

Supplementary table 4. Clinicopathological characteristics of patients with lung adenocarcinoma and associations with CD73 IHC expression in different membrane compartments of malignant cells

Characteristic		Total (T) CD73+ (79/106, 75%)			Basolateral (BL) CD73+ (68/106, 64%)			Tumors with evaluable Lumen	Luminal (L) CD73+ (60/72, 86%)		
	N	N	%	p value*	N	%	p value*		N	%	p value*
Age											
≤65	53	38	72%	0,6562	33	62%	0,8397	31	23	74%	0,1094
>65	53	41	77%		35	66%		41	37	90%	
Sex											
Female	52	41	79%	0,3759	39	75%	0,0268	37	31	84%	1
Male	54	38	70%		29	54%		35	29	83%	
Smoking History											
Never	15	15	100%	0,0107	13	87%	0,0788	15	15	100%	0,0598
Current/Former	91	64	70%		55	60%		57	45	79%	
TNM 8th Edition											
I	58	46	79%	0,2438	41	71%	0,0896	42	36	86%	0,6201
II	26	16	62%		12	46%		16	12	75%	
III	22	17	77%		15	68%		14	12	86%	
Pathological T (8th)											
pT1a - pT2a	70	54	77%	0,4810	47	67%	0,3984	50	43	86%	0,4932
pT2b - T4	36	25	69%		21	58%		22	17	77%	
Pathological N (8th)											
N0	78	59	76%	0,6922	51	65%	0,9400	55	46	84%	0,2996
N1	20	15	75%		12	60%		12	11	92%	
N2	8	5	63%		5	63%		5	3	60%	
Histologic pattern											
Any-Solid	46	29	63%	0,0243	29	63%	0,8412	12	10	83%	1
Non-Solid	60	50	83%		39	65%		60	50	83%	
Molecular characteristics											
EGFR Mutated	15	14	93%	0,1069	12	80%	0,2532	13	12	92%	0,4351
EGFR Wild-type	85	60	71%		54	64%		54	43	80%	
STK11 Mutated	7	3	43%	0,0626	3	43%	0,1948	7	3	43%	0,0041
STK11 Wild-type	56	44	79%		40	71%		35	33	94%	
KRAS Mutated	26	21	81%	0,6021	20	77%	0,1614	20	17	85%	1
KRAS Wild-type	77	56	73%		47	61%		50	42	84%	
TP53 Mutated	27	18	67%	0,2507	16	59%	0,2741	11	9	82%	0,6437
TP53 Wild-type	36	29	81%		27	75%		31	27	87%	
Somatic Mutation burden											
Median (range)	63	47	99 (2-955)	0.0400	43	127 (2-955)	0.5401	42	36	75 (2-940)	0.3178

Supplementary table 5. Overview of Luminal (L) and Basolateral (BL) membrane expression of CD73 in CD73 Groups.

Cell compartment CD73 IHC	T High N (%)	T low N (%)	T Negative N (%)	Total N (%)
BL+	28 (26.4)	35 (33.0)	1 (0.9)	64 (60.4)
BL–	0 (0)	16 (15.1)	26 (24.5)	42(39.4)
L+	13 (12.3)	51 (44.3)	0 (0)	60 (56.6)
L–	0 (0)	0(0)	12 (11.3)	12 (11.3)
L NE*	15 (14.2)	4 (3.7)	15 (14.2)	34 (32.8)
Total	28 (26.4)	51 (48.1)	27 (25.2)	106 (100)

*NE: not evaluable (no luminal membrane present)
